# How should we support young people with ASD and mental health problems as they navigate the transition to adult life including access to adult healthcare services

**DOI:** 10.1017/S2045796019000830

**Published:** 2020-01-09

**Authors:** Christopher King, Hannah Merrick, Ann Le Couteur

**Affiliations:** 1 Tees, Esk and Wear Valleys, NHS Foundation Trust, Tees, Esk and Wear Valleys, UK; 2 Population Health Sciences Institute, Newcastle University, Newcastle upon Tyne, UK

**Keywords:** Autism spectrum disorder, developmentally appropriate, transition, wellbeing

## Abstract

For young people with autism spectrum disorder (ASD), the transition from childhood to adulthood especially for those with additional mental health problems can be challenging. Increasing numbers of young people attending child and adolescent mental health services (CAMHS) have a recognised diagnosis of ASD. What are the outcomes of these young people when they are discharged from CAMHS and how best can services support their needs? In this editorial we consider the emerging literature on transition for young people with long-term conditions and in particular those with ASD. Longer term studies suggest that the outcomes for individuals with ASD across the ability range is mostly poor and that healthcare transfer has generally not been managed well, with service users often reporting a lack of appropriate types of support. Encouragingly there is an increasing awareness of the need to support young people with long-term conditions as they negotiate the many developmental tasks of transition to adulthood. However, less is known about the experiences and aspirations of autistic individuals of all abilities as they transition to adulthood. This knowledge can inform a more nuanced approach to identifying developmentally appropriate outcomes. Recent studies with cognitively able young people with ASD, highlight some features in common with young people with long-term conditions but also the importance of identifying ways to foster underlying skills and the ability of young people with ASD to develop and maintain relationships. Child-focussed and adult-orientated healthcare services need to work directly with autistic individuals and their support networks to facilitate successful engagement with services and enable adults to manage their mental health needs. There is an urgent need to investigate the implementation and effectiveness of research and clinical guideline recommendations that aim to increase wellbeing, health self-efficacy and improve the mental health outcomes for autistic adults.

## Introduction

Approximately 1% of the population (Williams *et al*., 2006; Elsabbagh *et al*., 2012) have a diagnosis of autism spectrum disorder (ASD). ASD in both childhood and adulthood is characterised by a spectrum of skills and needs, including impairments in social communication, and the presence of restricted and repetitive behaviours and interests (American Psychiatric Association, 2013). In addition, many individuals also meet criteria for co-occurring psychiatric conditions at a significantly higher rate compared to non-autistic populations (Simonoff *et al*., 2013). The conditions include attention deficit hyperactivity disorder (ADHD), anxiety and emotional disorders, and oppositional defiant disorder (Billstedt *et al*., 2005; Leyfer *et al*., 2006; Simonoff *et al*., 2013; Lever and Geurts, 2016). Not all individuals with ASD are diagnosed in childhood. Some individuals present to adult mental health services (AMHS) for an ASD diagnostic assessment, often with co-occurring physical and mental health needs (Brugha *et al*., 2012). There is an increasing recognition that primary health care (family practitioner) and community mental health services require the relevant expertise and resources to provide diagnostic assessment and intervention services appropriate for individuals of all ages presenting with a definite or suspected ASD diagnosis (Department of Health, 2015).

## Transition

Transition from childhood to adulthood is a period of development characterised by significant change of both the brain and behaviour (Colver and Longwell, 2013). The developmental tasks include leaving school, moving to post-secondary education or employment, gaining autonomy and independent living skills, and developing adult friendships and intimate relationships. The timescales vary, but the implication for services is that transition extends from approximately 16–24 years (Colver *et al*., 2018, 2019).

## Definitions for healthcare services

‘Transition’ refers to the purposeful, planned process that addresses the needs of young people in a holistic way as they move from child-centred to adult-oriented health care systems (Blum *et al*., 1993). In contrast, a ‘transfer’ is the singular event when medical care of a young person is moved from children's to adults' services. For example, the transfer from child and adolescent mental health services (CAMHS) to AMHS in the UK occurs at the age of 18 years (range 16–18 years).

## Transition experiences for young people with long-term conditions

Unfortunately, for many young people with long-term conditions during transition their health may deteriorate and social participation reduce. Studies of young adults with a range of disorders have demonstrated delays in autonomy, psychosexual and social development (Pinquart, 2014). These problems have been recognised internationally (NICE, 2016; Mazur *et al*., 2017; White *et al*., 2018). Publications highlight the importance of early planning, continuity of care and the need to consider developmental needs rather than simply defining an age cut-off healthcare transfer. The UK National Institute for Health and Care Excellence (NICE) guidelines recommend that: transition support must be developmentally appropriate and person-centred; young people should have a named worker and meet the adult team before transfer; support for building independence and appropriate involvement of parents/carers (NICE, 2016).

The evidence base supporting recommendations has been limited. A recently completed 5-year programme of applied health research on transition has attempted to address this knowledge gap. The programme included a 3-year longitudinal study of 374 young people with long-term conditions (diabetes, cerebral palsy and ASD with additional mental health problem) using generic outcomes such as subjective wellbeing, participation, satisfaction with services and condition-specific measures of disease control (Colver *et al*., 2019). Three features of transitional healthcare were associated with improved outcomes: appropriate parent/carer involvement (defined jointly by the young person and parent); promotion of health self-efficacy and meeting the adult team before transfer. Only 1/3 of young people across all groups experienced appropriate parental involvement; 1/4 of young people with ASD reported experiencing support with health self-efficacy and only a 1/4 had met the adult team (similar findings for young people with cerebral palsy) compared with two-thirds of young people with diabetes. The generalisability of these findings is limited as the sample was relatively young (17–21 years at final follow-up) and all were considered by their referring clinicians to be of average intellectual ability. However, the findings do provide sufficient evidence to support the urgent need to investigate how best to implement these relatively straightforward features of transitional healthcare. Other findings highlight the importance of organisation-wide training in ‘developmentally appropriate healthcare’; and the need for transitional healthcare to be funded and organised across child and adult healthcare providers, working in close partnership with primary care and community services. Implementation of these recommendations requires careful co-ordination within and between services – a definite challenge for existing funding organisations and service providers. The programme also included an exploration with young people of their attitudes to transition. Four broad approaches were identified: ‘laid-back’, ‘anxious’, ‘wanting to be in control’ and ‘socially-oriented’. These findings emphasise the need to individualise transition planning for each young person including consideration of their communication needs and skills.

### What are the transition experiences of young people who attend CAMHS?

Most young people with mental health problems in England and Wales attending CAMHS are discharged to primary care services rather than being referred to AMHS (Singh *et al*., 2010). For some, transfer of healthcare to primary care will be a positive and appropriate experience. For others, their mental health problems do not suddenly change, they cannot access an ongoing mental health service and experience a discontinuity of care provision (NICE, 2016). Accessing AMHS requires the young person to have an ‘enduring mental health problem’ (Singh *et al*., 2008). There is evidence from the UK Transition of Care from CAMHS to AMHS (TRACK) study that some young people with a range of mental health problems, including ASD, ADHD, emotional disorders and emerging personality disorders, have limited access to AMHS (Singh *et al*., 2010; Paul *et al*., 2013).

In a secondary analysis of the TRACK data, Islam *et al*. (2016) studied a subsample of 64 young people with ongoing mental health needs (just over a third of the original cohort) who were not transferred to AMHS. Twenty-three percent of this sample had a neurodevelopmental disorder. Whilst 11% were unsuccessfully referred, the majority had NOT been referred. A small proportion of cases were referred to other services, usually together with a referral to primary care. Over half of those who were not transferred to AMHS remained open to CAMHS services beyond the age for transition. The most common reason for non-referral was refusal by the young people/carer or resolution of the clinical need. Other reasons included disengagement from CAMHS; an assumption that the young people's mental health needs would not meet AMHS criteria; AMHS perceived to not have the required expertise; or transfer was delayed because of unknown immigration status. This study highlights the risk that young people with ASD, and others, may fail to access secondary mental health services and/or other relevant support at a time of increased uncertainty and risk.

The longitudinal Transition study (Colver *et al*., 2019) provided an opportunity to compare the experiences of young people with ASD attending CAMHS with those with diabetes and cerebral palsy. Healthcare transfer for 65% of young people with ASD was to primary care compared to over 90% of young people with diabetes who transferred to secondary adult healthcare services. At the beginning of the study young people and their parents (across all groups) were relatively satisfied with the services they were receiving. Over the course of the study the young people with cerebral palsy and with ASD became increasingly dissatisfied with services. Across all groups parents were usually more dissatisfied than young people (Colver *et al*., 2018). Despite this, the wellbeing of young people with diabetes (measured by the Warwick-Edinburgh mental well-being scale; Clarke *et al*., 2011) was within the average range for the general population. The scores for young people with cerebral palsy and ASD showed significantly poorer wellbeing, which persisted throughout the follow-up period. The young people with ASD had the lowest wellbeing scores by the end of the study.

The condition specific outcome measure for the ASD sample was the Hospital Anxiety and Depression Scale (HADS) – a 14 item self-report questionnaire designed to measure mental health symptoms in the week prior to the research visit (Zigmond and Snaith, 1983). An initial validation study of older adolescents and young adults with ASD has shown excellent psychometric properties (Uljarević *et al*., 2018). At each visit over the 3-years nearly 60% of young people had abnormal or borderline abnormal HADS anxiety scores.

A subsequent secondary analysis of outcome measures, follow-up visits and clinical notes was undertaken to investigate predictors of transfer to AMHS, and explore young peoples' experiences of transition (Colver *et al*., 2019). A diagnosis of ADHD and taking medication were the best predictors of transfer to AMHS. A thematic analysis identified seven categories: concerns about ASD/developmental and adolescent issues; engagement; family involvement; access to support services, educational and post-schooling opportunities; impact of mental health and crisis including self-harm. Using the young person's HADS scores three mental health trajectories were identified: doing well; continuing moderate difficulty and not doing well. A number of features were identified amongst the young people with the most positive outcomes including successful engagement by the young person (and their family) with services (school counsellors, social services and mental health services). Compared with those who had poorer outcomes, the young people in the ‘doing well’ group had experienced relative stability in educational provision and family life. There was also evidence that the young people were gaining skills in social participation, learning to manage their mental health concerns, developing an awareness of the impact of their ASD and learning to negotiate, with support, some of the developmentally appropriate aspects of transition. However, the young people who had successfully moved on to university did not meet criteria for the ‘doing well’ trajectory. They reported struggling to adjust to the challenging academic and social educational environments.

For some young people the discharge to primary care was not successful with crisis team involvement and time-limited support from AMHS before being discharged again. A common concern raised by families and young people was their perceived lack of support and ‘unmet need’ – much more common in the poorer outcome groups. These findings are in keeping with other research where young people of average ability are unable to access community learning disability, or other community specialist and mental health support. For all groups the importance of positive parent support was clear, with parents taking on roles co-ordinating support and in some cases providing employment opportunities. The poorer outcome groups were characterised by poor attendance and compliance with treatment, more significant impact of their ASD on progress, negative experiences with services leading to longer term disengagement, and young people not able to access services offered.

### Difficulties faced by young people with ASD

Prior to transition some young people with ASD experience bullying in school, especially those with more severe impairments in social skills, additional mental health problems and parents/carers who have mental health problems (Cappadocia *et al*., 2012). Following on from school, many face difficulties with low rates of post-secondary education and employment (Shattuck *et al*., 2012). Indeed, young adults aged 19–26 with ASD without intellectual disability (ID) are more likely than those with ID to have no daytime activities (Taylor and Seltzer, 2011; Taylor *et al*., 2015). Young people with ASD are reported to be less likely to be living independently (Howlin and Moss, 2012; Lake *et al*., 2014), are socially isolated (Lounds Taylor *et al*., 2017) and experience difficulties forming friendships and long-term relationships, including those who achieve a relatively high educational level (Hofvander *et al*., 2009). Autistic adults self-report high rates of loneliness (Hedley *et al*., 2018) and the experience of social disconnectedness and feeling a burden on others (Pelton and Cassidy, 2017). All these factors are likely to contribute to the high rates of comorbid psychiatric diagnoses (Taylor and Seltzer, 2011).

However, the broader social and economic context for young adults is changing, especially in developed economies, with more instability and uncertainty (Arnett, 2007; Wyn, 2014). These factors may also contribute to the anxiety expressed by parents (Sosnowy *et al*., 2018) and teachers (Elias *et al*., 2019) about the futures for autistic adults. Recent studies reporting the direct experiences of cognitively able autistic young people and adults provide new insights into their ambitions and transitional experiences *(*Cribb *et al*., 2019; Lambe *et al*., 2019). In the Sosnowy *et al*. (2018) study, although young people with ASD had some similar/equivalent long-term goals (e.g. finding post-secondary education or employment and/or leaving home), they focussed more on the steps towards achieving an outcome and/or gaining developmental precursor skills (e.g. understanding about how to make decisions for themselves). Cribb *et al*.'s (2019) qualitative study identified a group of young people seen in childhood and interviewed 12 years later. The thematic analysis identified that the young people (at age 16–20 years) felt more in control of their own lives, needed to take one step at a time and valued their ‘social connections with others’. These studies highlight the importance of the wider context for the young person and that acknowledging the pace of change is likely to promote wellbeing and a sense of self-identity. Minimising the impact of disruption of mental health provision or loss of support also appeared to promote continuation of underlying skills development and the ability of young people with ASD to make and maintain relationships.

In a mixed methods study Beresford *et al*. (2013) explored young people's and parents' experiences of transition planning and healthcare transfer. The lack of post-school options, especially amongst the cognitively able students ineligible for adult social care, and the lack of support caused greatest concern for parents and practitioners. For the authors, loss/interruption of mental health support at a time of change in so many areas of the young person's life appeared counter-intuitive. Cognitive ability was not an indicator of the degree in which the young person was able or willing to engage in planning and take on responsibility for moving forward towards adulthood. For many, involvement in planning was stressful and challenging. Further, the lack of daytime activities had a negative impact on wellbeing. Recommendations included the need for a ‘coordinator’ role to provide emotional support, anticipate preparatory skills and co-ordinate decision-making. Parents, not professionals, emerged as the most significant and valued source of support (Mitchell and Beresford, 2014).

### How can we better support young people with ASD through transition?

Young people with ASD have a wide range of skills and needs but as a group they can face multiple difficulties through childhood and into adulthood. These problems inevitably impact on achieving social independence. For autistic young people, individual functioning (including cognition, communication, independence and severity of ASD) is a strong predictor of outcome (Kirby *et al*., 2016). However, new evidence supports the need to appreciate skill differences as well as deficits. Differences in cognitive functioning might include social processing strengths and constructing personally significant narrative constructs, through to gender differences in mentalising all factors relevant to social outcomes. Further, it is important to appreciate what a ‘good outcome’ means for each young person and whether traditional outcomes of transition are developmentally appropriate, especially when the pressure of striving to achieve such social outcomes in the absence of support from adult services may be detrimental to wellbeing. Intervention programmes for autistic adults that focus on an individual's ability to develop social networks and friendships, and their underlying skills including planning and decision making, have been reported to lead to greater confidence in accessing social support (Oswald *et al*., 2018). These types of approaches may assist young people to manage their own health needs and learn how to navigate available service provision (health self-efficacy). However, most studies to date have not included young people with ID. Further research is needed to identify how best to appreciate the views and aspirations of this group.

There is an emerging evidence base that can inform healthcare funders and providers about the features of developmentally appropriate transitional care likely to be associated with improved outcomes for young people. This includes evidence about how best to organise commissioning (funding) and co-ordinate within and between agencies, including primary care, to improve transitional healthcare (Colver *et al*., 2019). The next step is to identify the most effective and efficient ways to implement these recommendations to improve social outcomes (Kirby *et al*., 2016; Maniatopoulos *et al*., 2018).

For autistic individuals access to adult mental health and other community support services is patchy and inadequate. There are calls for the establishment of autism treatment pathways in mental health services (Ward and Russell, 2007; Southby and Robinson, 2017; Camm-Crosbie *et al*., 2019). Although healthcare transfer is a single event, CAMHS and professionals in adult services need to support early preparation and planning with young people and their families to maximise opportunities for successful engagement with the young person, and work at an appropriate pace to develop their understanding of their own ASD and mental health needs. Being aware of the young person's immediate needs and reflecting positively on gains and achievements will help young people experience some control of their lives.

Using a regular self-report check such as the HADS may help young people, their families and the professionals supporting them, to identify the young person's trajectory, and the impact of individual and family life experience. This may promote discussion about ways to manage mental health needs that interfere with personal goals and achievements. Closer links between CAMHS, primary care and the emerging range of community adult mental health providers and other agencies is clearly necessary to reduce the risks that young people and families feel unsupported. For those young people and families who are overwhelmed by concerns about ASD, mental health problems and a sense that their needs are unmet, the risk is that they have a negative experience of transition, disengage with services, present in crisis and are unable to access any services and support offered to them. Perhaps the use of a tool such as the HADS might help identify those individuals especially at risk of negative outcomes and crisis presentations. With the pressure on CAMHS resources, identifying and prioritising the needs of those young people at greatest risk of poor outcomes might be of benefit if it allows clinicians to support these young people to identify their own transition goals for participation and future social relationships before they disengage with services and/or are discharged from CAMHS.
